# Markers Associated With Tumor Recurrence in Patients With Breast Cancer Achieving a Pathologic Complete Response After Neoadjuvant Chemotherapy

**DOI:** 10.3389/fonc.2022.860475

**Published:** 2022-04-20

**Authors:** Li-Yun Xie, Kun Wang, Hai-Lu Chen, Yan-Xia Shi, Yuan-Qi Zhang, Hao-Yu Lin, Yuan-Ke Liang, Ying-Sheng Xiao, Zhi-Yong Wu, Zhong-Yu Yuan, Si-Qi Qiu

**Affiliations:** ^1^Diagnosis and Treatment Center of Breast Diseases, Shantou Central Hospital, Shantou, China; ^2^Shantou University Medical College, Shantou, China; ^3^Department of Breast Cancer, Cancer Center, Guangdong Provincial People’s Hospital, Guangdong Academy of Medical Sciences, Guangzhou, China; ^4^Department of Medical Oncology, Sun Yat-sen University Cancer Center, Guangzhou, China; ^5^Department of Breast Surgery, Affiliated Hospital of Guangdong Medical University, Zhanjiang, China; ^6^Department of Thyroid and Breast Surgery, Clinical Research Center, The First Affiliated Hospital of Shantou University Medical College, Shantou, China; ^7^Guangdong Provincial Key Laboratory for Breast Cancer Diagnosis and Treatment, Shantou University Medical College, Shantou, China; ^8^Department of Thyroid Surgery, Shantou Central Hospital, Shantou, China; ^9^Clinical Research Center, Shantou Central Hospital, Shantou, China

**Keywords:** ALDH3A2, breast cancer, neoadjuvant chemotherapy, pathologic complete response, prognosis

## Abstract

**Background:**

Patients who achieve a tumor pathologic complete response (pCR) after neoadjuvant chemotherapy (NAC) have better outcomes than patients with residual tumor. However, tumors still recur in the pCR patients. Therefore, we aim to explore factors associated with tumor recurrence in this patient population.

**Methods:**

A total of 1,913 patients diagnosed with breast cancer between 1995 and 2020 and received NAC were included in this analysis. Clinicopathological data of the patients were retrospectively collected. We used Cox regression analysis to assess the associations of clinicopathological factors with patients’ outcome. Proteomic study of tumors was applied to identify differentially expressed proteins (DEPs) between tumors from the pCR patients with tumor recurrence and tumors from those without tumor recurrence. PPI network analysis of the corresponding genes of DEPs was used to identify the hub genes. The prognostic value of the corresponding genes of DEPs was evaluated using two online databases, Kaplan-Meier Plotter and bc-GenExMiner. The genes that were significantly associated with patients’ survival in both databases, as well as being identified as hub genes, were considered as potential prognostic markers for pCR patients. Publicly available data from Gene Expression Omnibus (GEO) was used to verify the prognostic value of the identified marker.

**Results:**

Among the 1,913 included patients, 420 had tumor pCR. The median follow-up for the pCR patients was 32.6 months (IQR, 16.3-55.5). Overall estimated 5-year risk of tumor recurrence for the pCR patients was 11%. Multivariable analysis showed that a higher pre-NAC clinical T stage and N stage were independent predictors for increased risk of tumor recurrence (hazard ratio [HR] 2.57, 95% confidence interval [CI] 1.01-6.51, P=0.047 for clinical T stage and HR 3.48, 95%CI 1.37-8.83, P=0.009 for clinical N stage). NAC regimens, the type of breast and axillary surgery, and adjuvant chemotherapy were not associated with tumor recurrence. Finally, aldehyde dehydrogenase (ALDH) 3A2 was identified by the proteomic study and was verified as a potential predictor for tumor recurrence in the pCR patients (with a median follow up of 3.78 years for dataset GSE32603 and 2.74 years for dataset GSE25066 from GEO, tumor recurrence rate: low versus high expression, 20.7% versus 4.5% [data from GSE32603]; 10.9% versus 0% [data from GSE25066]).

**Conclusions:**

Clinical T stage, clinical N stage and tumor expression of ALDH3A2 were potential markers for predicting tumor recurrence in the pCR patients after NAC.

## Introduction

Neoadjuvant chemotherapy (NAC) is widely used to downstage breast cancer to increase the rates of breast conserving surgery as well as to reduce the extent of axillary surgery (1). As the tumor remains intact during the systemic treatment, NAC allows monitoring of treatment response. The CTNeoBC pooled analysis and several randomized trials, such as NeoSphere and TRYPHAENA, have demonstrated that 17%-66% of the patients achieved tumor pathologic complete response (pCR) after NAC (2–4). The varied pCR rates reported from the above-mentioned studies largely depend on the differences in molecular subtypes of breast cancer included and the different therapeutic regimens used in these studies. For example, the additional pertuzumab to the conventional regimen has significantly increased the pCR rate (3, 5). Importantly, compared with the patients with residual tumors, the pCR patients have achieved superior long-term survival, especially for those with human epidermal growth factor receptor 2 (HER2) positive and triple negative breast cancers (4–7). Therefore, pCR has been commonly used as an endpoint for assessing the treatment efficacy in clinical trials regarding NAC (8).

Nevertheless, despite improved survival is observed in the pCR patients, a proportion of these patients will finally develop breast cancer recurrence (4–6, 9). This emphasizes the potential necessity of adjuvant treatment escalation after NAC for a subgroup of pCR patients to reduce the tumor recurrence risk, although the tumor is eradicated by NAC. The strategy of adjuvant treatment escalation has successfully improved the patients’ outcome in the patients with residual tumors after NAC (10, 11). However, this treatment strategy is not standard-of-care in current practice for the pCR patients, due to the lack of solid evidence from randomized controlled trails. Therefore, revealing of the risk factors of tumor recurrence in the pCR patient population would be helpful for identification of a subgroup with high-risk of tumor recurrence, and subsequently supporting exploration of adjuvant treatment escalation in the high-risk patients.

Therefore, we aimed to explore clinicopathological factors associated with tumor recurrence in the pCR patients, using data of a large cohort of patients treated with NAC in four university hospitals from Southern China. Furthermore, proteomics study was used to identify potential prognostic biomarkers in this patient population. Finally, we used publicly available data from the Gene Expression Omnibus (GEO) database to verify the prognostic value of the identified biomarker.

## Materials and Methods

### Patients and Samples

In this retrospective analysis, female patients aged ≥18 years and diagnosed with breast cancer and received NAC between 1995 and 2020 were eligible. Patients with initially metastatic disease (stage IV), bilateral breast cancer or no surgical treatment for breast cancer were excluded from the present study. A total of 1,913 patients were identified in four university hospitals and included for the analysis in this study, including 1,037 patients from Sun-Yat Univeristy Cancer Center, 102 patients from Shantou Central Hospital who participated in a multicentre phase III trial (NCT03006614), 113 patients from Central People’s Hospital of Zhanjiang, and 661 patients from Guangdong Provincial People’s Hospital. Out of the 1,913 patients, 420 achieved a tumor pCR. Information on patient, treatment and tumor characteristics were collected from the electronic medical records systems in the four participating hospitals. These information include age at diagnosis, type of breast surgery and axillary surgery, pre-NAC clinical T stage and clinical N stage of tumor, histological subtype and grade of tumor, estrogen receptor (ER) status, progesterone receptor (PR) status, HER2 status and Ki67 expression. Positivity of ER, PR and HER2 were determined according to the American Society of Clinical Oncology/College of American Pathologists (ASCO/CAP) guidelines (12, 13). Briefly, ER and PR were considered positive if there were at least 1% positive tumor nuclei in the tumor sample being observed (12). HER2 was considered positive if there was evidence of protein overexpression (immunohistochemistry staining 3+) or gene amplification (fluorescent *in situ* hybridization with a HER2/CEP17 ratio ≥2 or average HER2 copy number ≥ 6 signals/cell) (13). Low level of Ki67 expression was reported as percentage of cells with positive nuclei staining < 20% and high level of Ki67 expression as ≥ 20%. pCR was defined as no evidence of invasive cancer in the breast and axillary nodes, irrespective of ductal carcinoma *in situ* (ypT0/is/ypN0). Among the pCR patients from Guangdong Provincial People’s Hospital, 13 had tumor recurrent event. Matched tumor-free patients were selected according to patient’s age at diagnosis, tumor grade, clinical T stage, clinical N stage, ER, PR and HER2 status. Eventually, 7 samples from patients with recurrent events and 5 samples from matched patients without any events were available and used for proteomics study. According to the regulations of the Ethical Committees in the four participating hospitals, this retrospective non-interventional study did not require informed consent from the studied patients.

### Protein Identification and Quantification

Label-free quantification method was used to determine the relative amount of proteins in the 12 samples (7 and 5 from event and event-free groups, respectively). Raw data were submitted for analysis in Proteome Discoverer v2.2 (PD 2.2, Thermo) software. The protein quantitation results were statistically analyzed by Mann-Whitney U Test. Proteins whose quantitation were significantly different between the event and event-free groups (defined as FC > 1.2 or FC < 0.83 and P-value < 0.05, event group versus event-free group), were defined as differentially expressed proteins (DEPs). In addition, corresponding genes of DEPs were defined as differentially expressed genes (DEGs). Details on protein isolation processing are provided in the Supplemental Appendix (**Supplementary File 1**).

### Gene Ontology and and Kyoto Encyclopedia of Genes and Genomes Enrichment Analyses of DEPs

To analyze the potential biological mechanisms influencing the prognosis of the pCR patients, the DEPs were applied to Gene Ontology (GO) and Kyoto Encyclopedia of Genes and Genomes (KEGG) pathway enrichment analyses using the clusterProfiler package in R. GO enrichment analysis was used to annotate the DEPs meaningfully in terms of their biological processes, cellular components and molecular functions. KEGG enrichment analysis was performed to analyze the biological pathways of the target DEPs.

### PPI Network and Hub DEGs

The PPI network of the DEGs was constructed by STRING v11.5 (http://string-db.org/). Then the PPI network was subjected to the Network Analyzer tool of Cytoscape v3.8.2 and cytoHubba plugin, where the degree of genes was calculated as the direct number of edges linking to a given node gene. The top 10 genes in terms of their degree of connectivity were defined as the hub genes.

### Identification of Potential Biomarkers Associated With Tumor Recurrence

DEPs with FC > 2.0 or FC < 0.5 were selected in order to select proteins with a larger difference in their expression level between the event and the event-free patients. A total of 40 DEPs were identified. The prognostic value of their corresponding gene expression was evaluated using two online databases, Kaplan-Meier Plotter (http://www.kmplot.com) and bc-GenExMiner v4.7 (http://bcgenex.ico.unicancer.fr), which contained gene expression data and survival information of breast cancer patients. The median expression level of the genes was used as cutoff to defined the high expression and the low expression levels. Data of recurrence-free survival (RFS) and distant metastasis-free survival (DMFS) of patients were obtained from the Kaplan-Meier Plotter and bc-GenExMiner, respectively. The genes that were significantly associated with patients’ survival in both databases, as well as being identified as hub genes in the above-mentioned PPI network analysis, were considered as potential prognostic biomarkers for pCR patients.

### Verification of the Prognostic Value of Identified Biomarkers Using Publicly Available Database

In order to verify the prognostic value of identified biomarkers in patients treated with NAC, the patient data and corresponding tumor gene expression data of two NAC databases (GSE32603 and GSE25066) were obtained from the GEO database. RFS and DMFS of patients were presented in groups classified by median expression of the identified genes (high versus low expression). The distribution of the RFS and DMFS events according to the expression level of the identified biomarkers was demonstrated in the pCR and non-pCR patients, respectively, using the Stack bar chart.

### Statistical Analysis

The continuous variables were described by median and interquartile range (IQR) and the categorical variables were described by percentages. RFS was defined as the time between the date of surgery and the date of disease recurrence. DMFS was defined as the time between the date of surgery and the date of the occurrence of distant metastases. The remaining patients were censored at the date known to have no recurrent events. The prognostic value of clinicopathological factors was determined using univariable and multivariable Cox regression analyses. Variables with a P value ≤ 0.05 in the univariable analysis were included in the multivariable analysis. We used the list-wise deletion method for handling the missing data. With this method, we excluded the entire sample from the multivariable Cox regression analysis if any single value was missing for the variables. RFS and DMFS were analyzed using the Kaplan–Meier survival analysis. A log-rank test was used to assess its difference. P values tested two-sided ≤ 0.05 were considered significant. Statistical analysis was performed using the Statistical Package for the Social Sciences (SPSS) v19.0 (SPSS. Inc.). Data were plotted using the GraphPad Prism v8.3.0 (GraphPad Software. Inc).

## Results

### Patient and Tumor Characteristics

**Figure 1:**
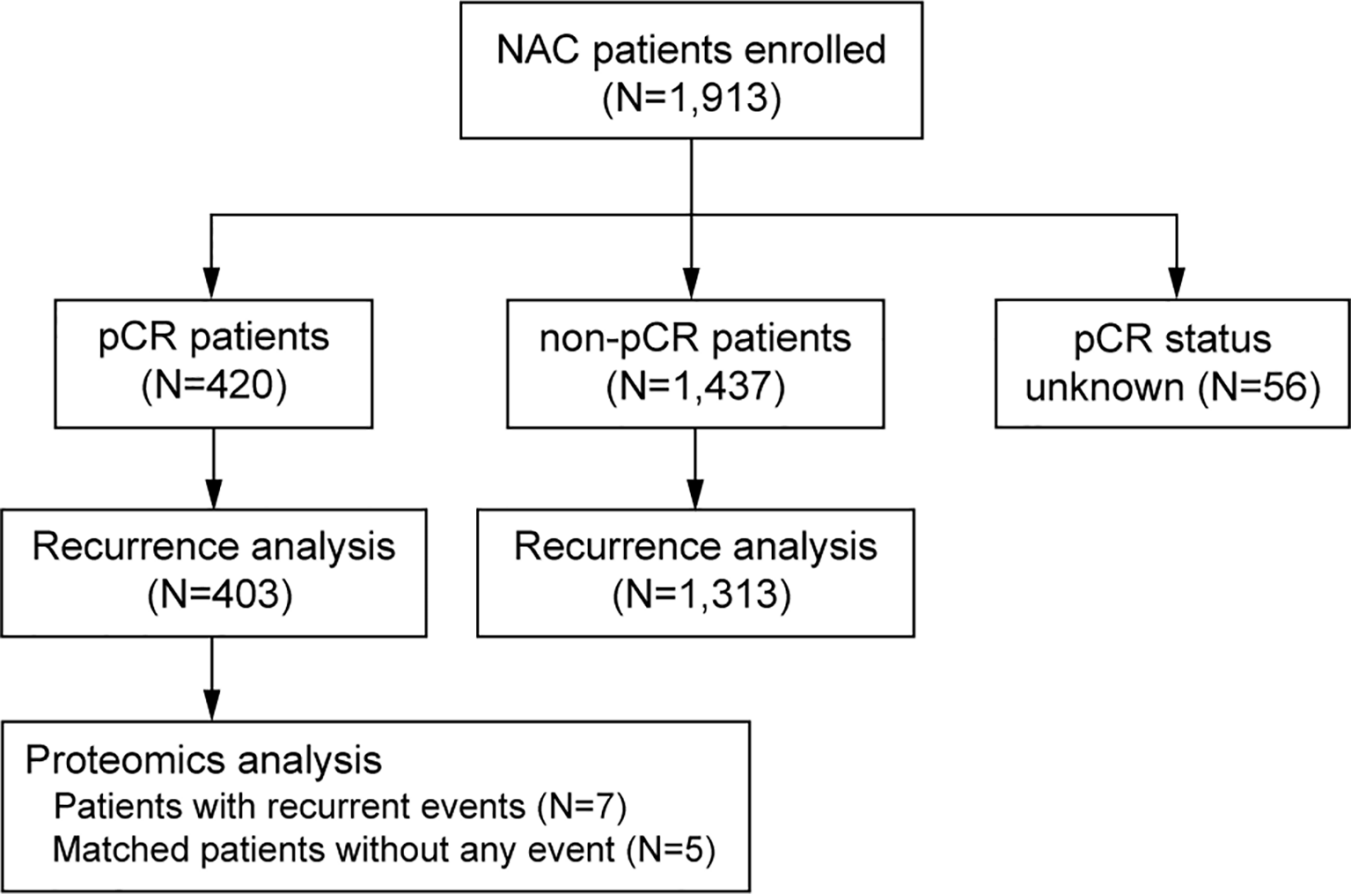
CONSORT diagram of the studied patients, number of patients with different pCR status, number of patients for survival analysis and number of patients for proteomics analysis. pCR, pathologic complete response.

A total of 1,913 identified patients were included for analysis (**Figure 1**). Patient and tumor characteristics of the patients are shown in **Table 1** and **Supplementary Table 1**. The median age at diagnosis of breast cancer was 48 years (IQR, 41-55). Most of the patients had clinical T2-T3 tumors (76.4%), and N1-N2 tumors (65.3%). More than 60% of the patients had ER-positive tumors. PR positivity was observed in 55.5% of the cases, and HER2 positivity in 43.9% of the patients. High level of Ki67 expression was present in 81.6% of the tumors. Tumor pCR was observed in 22.6% of the studied patients.

**Table 1:** Clinicopathological characteristics of the 1,913 patients.

Characteristics	No. (%)	(%) exclude unknown
**pCR status**		
non-pCR	1,437 (75.1)	(77.4)
pCR	420 (22.0)	(22.6)
Unknown	56 (2.9)	
**Age at diagnosis**		
(median, IQR)	48 (41-55) years	
**Age**		
≤50	1,141 (59.6)	(59.6)
>50	772 (40.4)	(40.4)
**Clinical T stage**		
T1	163 (8.5)	(9.5)
T2	916 (47.9)	(53.4)
T3	394 (20.6)	(23.0)
T4	243 (12.7)	(14.2)
Unknown	197 (10.3)	
**Clinical N stage**		
N0	333 (17.4)	(19.8)
N1	521 (27.2)	(31.0)
N2	577 (30.2)	(34.3)
N3	249 (13.0)	(14.8)
Unknown	233 (12.2)	
**Clinical TNM stage**		
I	61 (3.2)	(3.6)
II	609 (31.8)	(36.3)
III	1,006 (52.6)	(60.0)
Unknown	237 (12.4)	
**Histological type**		
Invasive cancer non-specified	1,519 (79.4)	(93.5)
Others	106 (5.5)	(6.5)
Unknown	288 (15.1)	
**Histological grade**		
I	30 (1.6)	(2.3)
II	698 (36.5)	(54.2)
III	561 (29.3)	(43.5)
Unknown	624 (32.6)	
**ER**		
Negative	673 (35.2)	(37.1)
Positive	1,142 (59.7)	(62.9)
Unknown	98 (5.1)	
**PR**		
Negative	790 (41.3)	(44.5)
Positive	986 (51.5)	(55.5)
Unknown	137 (7.2)	
**HER2**		
Negative	986 (51.5)	(56.1)
Positive	771 (40.3)	(43.9)
Unknown	156 (8.2)	
**Ki67**		
<20%	327 (17.1)	(18.4)
≥20%	1,447 (75.6)	(81.6)
Unknown	139 (7.3)	
**Molecular subtype**		
HR+/HER2-	710 (37.1)	(40.6)
HER2+	771 (40.3)	(44.1)
TNBC	267 (14.0)	(15.3)
Unknown	165 (8.6)	
**NAC regimen**		
E/T/E+T	1,202 (62.8)	(63.9)
Others	678 (35.4)	(36.1)
Unknown	33 (1.7)	
**Breast surgery**		
BCS	305 (15.9)	(16.3)
Mastectomy	1,561 (81.6)	(83.7)
Unknown	47 (2.5)	
**Axillary surgery**		
SLNB	334 (17.5)	(17.6)
ALND+/-SLNB	1,563 (81.7)	(82.4)
Unknown	16 (0.8)	
**Tumor recurrence**		
No	1,323 (69.2)	(72.4)
Yes	504 (26.3)	(27.6)
Unknown	86 (4.5)	
**Recurrent or metastatic lesions**		
Local-regional relapse	209 (10.9)	(11.4)
Liver	63 (3.3)	(3.5)
Lung	74 (3.9)	(4.1)
Bone	98 (5.1)	(5.4)
Brain	28 (1.5)	(1.5)
Soft tissue	28 (1.5)	(1.5)
Ovary	1 (0.1)	(0.1)
Multi-organs	103 (5.4)	(5.6)
Unknown	1 (0.1)	

AC, adjuvant chemotherapy; ALND, axillary lymph node dissection; BCS, breast-conserving surgery; E, antharcycline; ER, estrogen receptor; HER2, human epidermal growth factor receptor 2; IQR, interquartile range; NAC, neoadjuvant chemotherapy; PR, proges-terone receptor; SLNB, sentinel lymph node biopsy; T, taxine.

### Prognosis of pCR Patients Was Better Than That of the Non-pCR Patients

**Figure 2:**
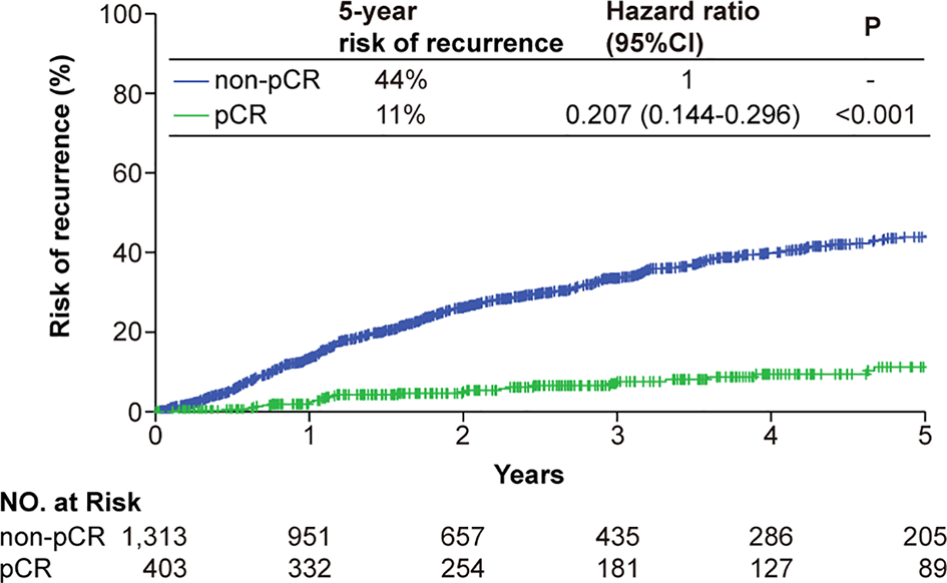
Kaplan-Meier analysis of the risk of tumor recurrence classified by the pCR status. Patients achieving a tumor pCR had a significantly lower risk of tumor recurrence. Abbreviations: CI, confidence interval; pCR, pathologic complete response.

The median follow-up of RFS for the entire study population was 24.9 months (IQR, 11.6-46.0 months). The pCR status was correlated to RFS in the univariable Cox regression analysis, and remained significant in the multivariable analysis (**Supplementary Figure 1**). The pCR patients had a significantly improved RFS compared with that of the non-pCR patients (hazard ratios [HR], 0.21, 95% confidence interval [CI] 0.14-0.30) (**Figure 2**). Similar trend of superior RFS in the pCR patients was observed across all subgroups in the subgroup analysis (**Supplementary Figure 2**).

### Clinicopathological Factors Associated With RFS in The pCR patients

**Figure 3:**
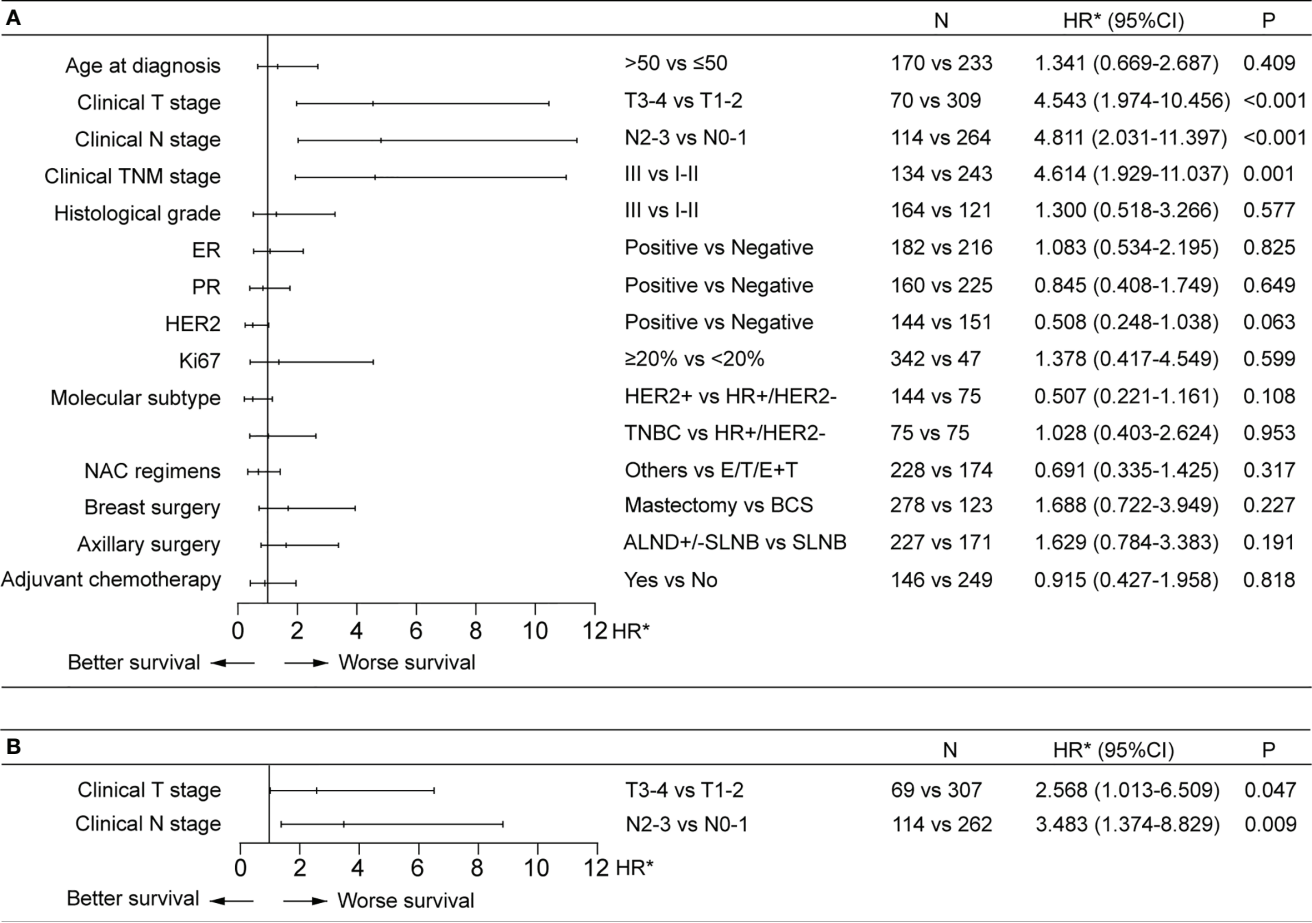
Factors associated with recurrence-free survival (RFS) in univariable **(A)** and multivariable **(B)** Cox regression analysis in the pCR patients. **(A)** Higher pre-NAC clinical T and clinical N stages were associated with worse RFS in the univariable Cox regression analysis. **(B)** Higher pre-NAC clinical T and clinical N stages were independent prognostic factors for RFS. ALND, axillary lymph node dissection; BCS, breast-conserving surgery; CI, confidence interval; E, anthracycline; ER, estrogen receptor; HR, hormone receptor; HR*, hazard ratio; HER2, human epidermal growth factor receptor 2; NAC, neoadjuvant chemotherapy; PR, progesterone receptor; SLNB, sentinel lymph node biopsy; T, taxine; TNBC, triple-negative breast cancer.

The median follow-up for the 420 patients achieving a pCR was 32.6 months (IQR, 16.3-55.5 months). Out of these 420 patients, 32 eventually developed cancer recurrence. The estimated 5-year risk of tumor recurrence was 11% (**Figure 2**). Higher pre-NAC clinical T stage and clinical N stage were associated worse RFS in the univariable Cox regression analysis, as well as in the multivariable analysis, being independent predictors of worse RFS (HR 2.57, 95%CI 1.01-6.51 for clinical T stage and HR 3.48, 95%CI 1.37-8.83 for clinical N stage) (**Figure 3**). NAC regimens, the type of breast and axillary surgery, and adjuvant chemotherapy were not associated with tumor recurrence (**Figure 3**).

### DEPs Between the pCR Patients with a Recurrent Event and Matched Patients Without an Event

**Figure 4:**
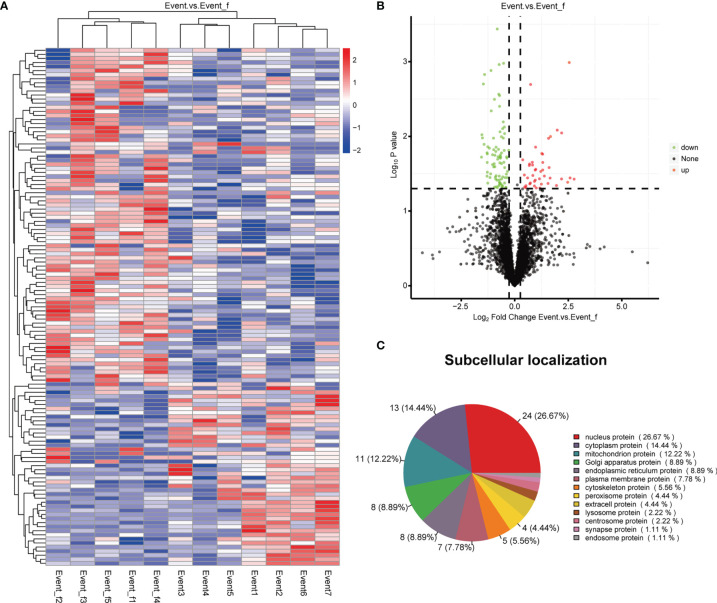
Heatmap, volcano plot and subcellular localization pie chart of the differentially expressed proteins (DEPs). **(A)** Heatmap of the DEPs. **(B)** Volcano plot of the DEPs. **(C)** Subcellular localization pie chart of the DEPs.

A total of 127 proteins were identified as DEPs, including 43 up-regulated and 84 down-regulated proteins. A heatmap and a volcano plot of these DEPs are shown in **Figure 4**. In terms of their subcellular localization, more than a quarter of the DEPs were nucleus proteins, about 14% were cytoplasm proteins, and 12% were mitochondrion proteins (**Figure 4C**). All DEPs and their corresponding genes are listed in **Supplementary Table 2**.

**Figure 5:**
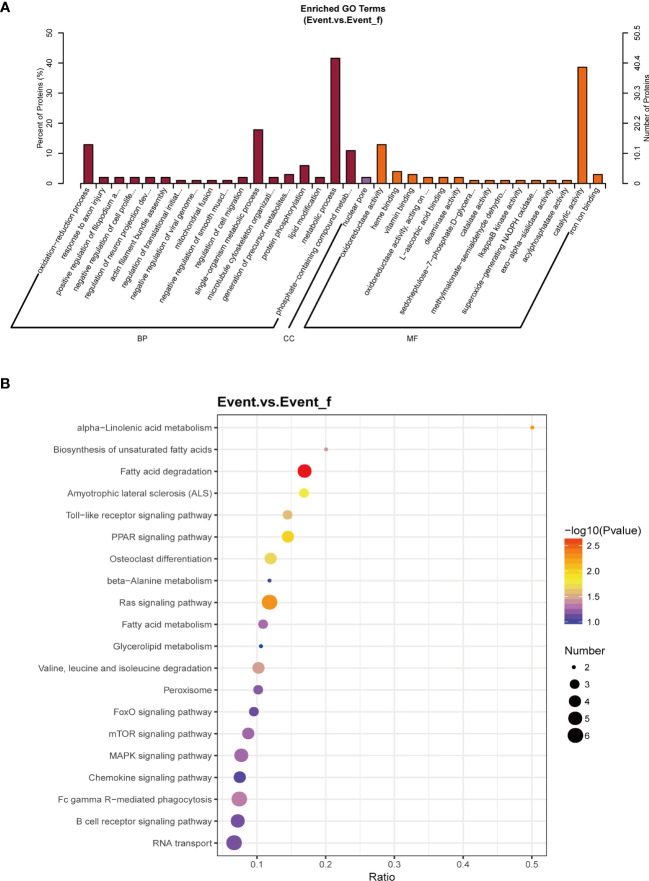
The biological functions of all differentially expressed proteins (DEPs). **(A)** Gene Ontology (GO) enrichment analysis of the DEPs. **(B)** Kyoto Encyclopedia of Genes and Genomes (KEGG) enrichment analysis of the DEPs. The main signal pathways the DEPs involved in including pathways related to lipid metabolism. BP, biological process; CC, cellular component; Event_f, event free; MF, molecular function.

To further explore the biological functions of the DEPs, GO function enrichment analysis and KEGG pathway enrichment analysis were performed on the DEPs. The enriched GO terms are demonstrated in **Figure 5A**, including the biological processes (BP) like metabolic process and oxidation-reduction process, and the molecular functions (MF) such as catalytic activity and oxidoreductase activity. The main signal pathways the DEPs involved in including pathways related to lipid metabolism, such as fatty acid degradation and biosynthesis of unsaturated fatty acids (**Figure 5B**). GO and KEGG enrichment analyses in the up-regulated and down-regulated proteins are showed separately (**Supplementary Figures 3**, **4**).

### Identification of Biomarkers Associated With Tumor Recurrence in the pCR Patients

**Figure 6:**
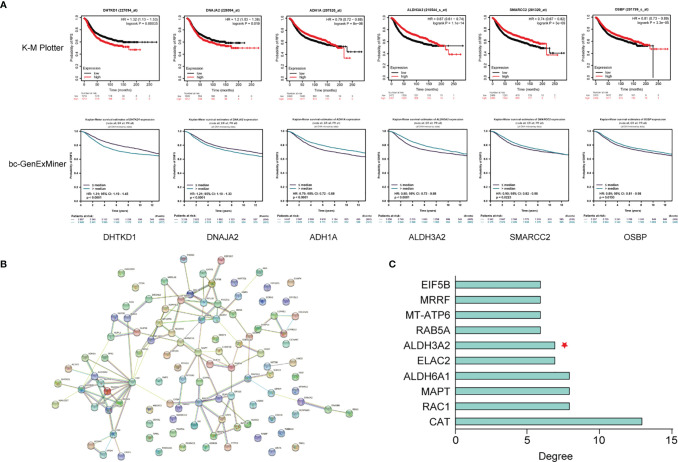
Identification of ALDH3A2 as a potential biomarker associated with tumor recurrence in the pCR patients. **(A)** Survival analysis of six differentially expressed genes (DEGs) in K-M Plotter and bc-GenExMiner databases. **(B)** The PPI network of the DEGs. **(C)** Hub genes of the DEGs. K-M Plotter, Kaplan-Meier Plotter; PPI network, protein-protein interaction network.

Among the corresponding genes of the 40 DEPs with FC > 2.0 or FC < 0.5, six genes (DHTKD1, DNAJA2, ADH1A, ALDH3A2, SMARCC2 and OSBP) were significantly associated patient prognosis in both two publicly available databases (**Figure 6A**). The PPI network of the DEGs is shown in **Figure 6B** and the hub genes with degree of connectivity in the top 10 list in **Figure 6C**. ALDH3A2 was selected as a potential prognostic biomarker for the pCR patients, as this gene both demonstrated prognostic value in the above-mentioned survival analyses and was identified as a hub gene.

### Verification of ALDH3A2 as a Biomarker Associated With Tumor Recurrence in the pCR Patients

**Figure 7:**
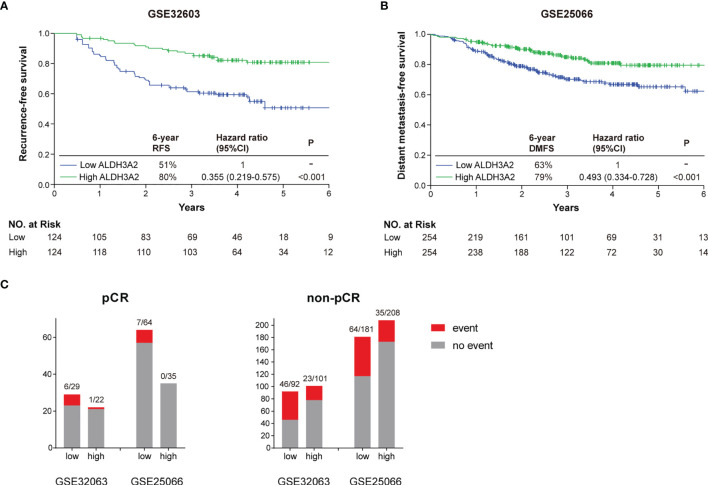
Verification of ALDH3A2 as a biomarker associated with tumor recurrence in the pCR patients. **(A)** Kaplan-Meier analysis of recurrence-free survival classified by the ALDH3A2 tumor expression in GSE32603. **(B)** Kaplan-Meier analysis of distant metastasis-free survival classified by the ALDH3A2 tumor expression in GSE25066. **(C)** Distribution of the recurrent events according to the ALDH3A2 tumor expression in the pCR and non-pCR patients from the GSE32603 and GSE25066 datesets. CI, confidence interval; DMFS, distant metastasis-free survival; RFS, recurrence-free survival.

To verify the prognostic value of ALDH3A2 tumor expression, we performed survival analyses on ALDH3A2 using two publicly available independent datasets from the GEO database (GSE32603 and GSE25066). These two datasets included 148 and 508 breast cancer patients treated with NAC, respectively. With a median follow up of 3.78 years for GSE32603 and 2.74 years for GSE25066, we found a significantly lower risk of tumor recurrence in patients with high expression of ALDH3A2 in the primary tumor compared with those having tumors with low expression of ALDH3A2 in both databases (**Figure 7**). A total of 150 patients had a tumor pCR from the two databases, with 51 patients from GSE32603 and 99 patients from GSE25066. Among these patients, 14 eventually developed tumor recurrence. Out of these 14 patients, 13 had tumors with low expression of ALDH3A2 (**Figure 7C**). Additionally, low expression of ALDH3A2 was associated with a higher risk of tumor recurrence in the non-pCR patients (**Figure 7C**).

## Discussion

In this multicentre study, we demonstrated that pre-NAC clinical T and clinical N stage were independent predictors of tumor recurrence for the pCR patients after NAC. In addition, we identified ALDH3A2 as a potential prognostic biomarker for this patient population. To our knowledge, this is the first study to show an association between a lipid metabolism associated factor and the risk of tumor recurrence in pCR patients.

The rate of tumor recurrence (11%) we observed in our study was similar with what has been previously reported (10-25%) (14–17). Previous studies mainly assessed the prognostic value of clinicopathological factors in pCR patients (14–20). Higher clinical stage has been shown to be associated with a higher risk of tumor recurrence in these studies (14, 15, 18–20). Similarly, our results demonstrated that the pCR patients with higher clinical T or clinical N staged tumors had a poorer outcome. Together, these results indicate a need of post-surgery intensive surveillance to enable early detection of tumor recurrence in the pCR patients with a higher pre-NAC clinical stage. The prognostic value of HER2 positivity in the pCR patients was inconclusive from the literature (17, 19, 20). Asaoka et al. and Tanioka et al. identified HER2 positivity as a predictor of worse patient outcome (17, 20). In our study, HER2 positivity showed no prognostic significance in patients who had achieved a pCR. This result is consistent with findings from a recent study by Chaudry et al. (19). One possible explanation for the different prognostic value of HER2 positivity among these studies is the difference in the percentage of patients received anti-HER2 targeted therapy. In the study by Chaudry et al. and our study, 80% of the patients with HER2-positive tumors received anti-HER2 therapy, compared with 63% of the patients in the study by Tanioka et al. and thus a higher percentage of patients did not get benefit from the anti-HER2 therapy (17, 19).

Interestingly, in the present study neither NAC regimen nor adjuvant chemotherapy was associated with tumor recurrence. These results are consistent with findings from the literature (21, 22). In a study of 721 patients who achieved pCR after NAC, compared with patients received adriamycin-based (A) chemotherapy plus taxane (T) in the NAC setting, the RFS was not significantly different in patients with A without T and in those with HER2-targeted therapy (21). In a meta-analysis of 52 studies including a total of 27,895 patients, the outcome was similar between patients with and without adjuvant chemotherapy who attained pCR after NAC (22). For HER2-positive patients who achieved pCR after neoadjuvant pertuzumab plus trastuzumab (PH), the 3-year event-free survival (EFS) rates were comparable between those received trastuzumab (PH→H) and pertuzumab plus trastuzumab (PH→PH) in the adjuvant settings, being 92% (95% CI: 87%–95%) and 95% (95% CI: 90%–97%) respectively (23). However, the 3-year EFS was much lower in patients who received trastuzumab in both neoadjuvant and adjuvant settings (H→H; 87% [95% CI: 82%–90%]) (23). These results likely reflect tumor biology and provide some reassurance for designing prospective trials aiming at de-escalation of adjuvant treatment for part of the pCR patients, such as those with a low risk of tumor recurrence.

To our knowledge, this is the first study to evaluate the impact of the type of surgery on tumor recurrence in pCR patients. Neither the type of breast surgery nor the type of axillary surgery demonstrated any prognostic significance in our study. pCR is reflective of eradication of tumors in both the breast and the axilla by NAC. Therefore, theoretically, it may be reasonable to omit the breast or axilla surgery and adopt a “watch-and-wait” approach if a tumor pCR is achieved. The difficulty remained is how to accurately predict the pCR status. Regretfully, in general the false-negative rates of nonsurgical tools such as imaging or minimally invasive image-guided multiple biopsy are too high to be as reliable as surgery (24–27). It is worthy but challenging to conduct clinical trials to develop reliable methods for selecting a subgroup of patients who derive no benefit from surgery after NAC.

ALDH has been used in many studies as a biomarker of stem-like cancer cells and aggressive tumor behavior in solid tumors including breast cancer (28, 29). There is accumulating evidence suggesting that study on the isoforms of ALDH would be crucial when examining its role as a stem cell biomarker or in promoting cancer metastasis (29–32). ALDH3A2, which is in the ALDH family 3, is a detoxifying enzyme that oxidizes the long-chain aliphatic aldehydes to fatty acids to reduce injury of lipid peroxidation (33). In a recent study on acute myeloid leukemia (AML), ALDH3A2 protected AML cells from oxidative death (34). Depletion of Aldh3a2 induced ferroptotic cell death in leukemic cells and was synthetically lethal with the treatment of a ferroptosis inducer, glutathione peroxidase-4 (GPX4) inhibitor RSL3 (34). The role of ALDH3A2 may be different in solid tumors. High expression of ALDH3A2 in the tumor tissue has been shown to be associated with prolonged overall survival in patients with gastric cancer and clear cell renal cell carcinoma (35, 36). In prostate cancer, the expression of ALDH3A2 was decreased in the primary cancer tissue compared to the healthy prostate tissue but increased in response to anticancer treatments (37). Similarly, the expression of ALDH3A2 was downregulated in gastric cancer tissue compared to normal tissue (38). Ours is the first study to evaluate the prognostic role of ALDH3A2 in breast cancer. We found that high expression of ALDH3A2 was associated with a lower risk of tumor recurrence in patients received NAC, including those achieved a tumor pCR. Together, these findings potentially indicate a tumor suppressor role of ALDH3A2 in solid tumors. Although the underlying mechanisms are poorly understood, ALDH3A2 may have an impact on the infiltration of immune cells and may also affect the expression of immune checkpoints (35). The prognostic value and biological functions of ALDH3A2 in solid tumors therefore deserve further investigation.

Our study has limitations. First, due to the retrospective nature, part of the data on patient and tumor characteristics was missing. However, the number of patients excluded from the recurrence analysis was limited (with 403 out of 420 patients included for the analysis), and therefore, a major impact on our findings would be unlikely. Second, the follow up for the pCR patients was relatively short, considering the excellent patient outcome in this patient population. This resulted in a small number of patients with recurrent events. Therefore, the results of this study will be validated in an ongoing study with a much larger number of patients from 22 hospitals, as well as with a longer follow up period. The prognostic value of DHTKD1, DNAJA2, ADH1A, ALDH3A2, SMARCC2 and OSBP in pCR patients were not assessed in this study. This would be interesting to be evaluated in future studies.

In conclusion, patients who achieve a tumor pCR after NAC have a much lower risk of tumor recurrence compared with those without pCR. However, we observed that among the pCR patients, those with higher pre-NAC clinical T and clinical N stage have a higher risk of tumor recurrence. In addition, we for the first time identified ALDH3A2 as a potential prognostic biomarker for this patient population.

## Data Availability Statement

The original contributions presented in the study are included in the article/**Supplementary Material**. Further inquiries can be directed to the corresponding authors.

## Ethics Statement

The studies involving human participants were reviewed and approved by Medical Ethics Committee of Shantou Central Hospital. Written informed consent for participation was not required for this study in accordance with the national legislation and the institutional requirements.

## Author Contributions

L-YX, KW and H-LC designed the study, performed the data acquisition, analyzed and interpreted the data, and drafted the manuscript. Y-XS, Y-QZ and Y-SX performed the data acquisition, interpreted the data, and drafted the manuscript. H-YL and Y-KL interpreted the data and critically revised the manuscript. Z-YW supervised parts of the study and critically revised the manuscript. Z-YY and S-QQ designed and supervised the study, interpreted the data, and drafted the manuscript. All authors contributed to the article and approved the submitted version.

## Funding

This study is supported by the Natural Science Foundation Committee (81901801 and 82102948), Natural Science Foundation of Guangdong Province (2021A1515011180, 210715106900933 and 2021A1515012178), Science and technology projects of Shantou (200623115260299), Shantou Central Hospital Research Incubation Program (201905), Interdisciplinary project of Li-Ka-Shing Foundation (2020LKSFG05C and 2020LKSFG04A). The funding bodies did not participate in the design of the study; the collection, analysis, or interpretation of the data; the writing of the manu-script; or the decision of manuscript submission. Wu JiePing Medical Foundation (Clinical Research Special Project, 320.6750).

## Conflict of Interest

The authors declare that the research was conducted in the absence of any commercial or financial relationships that could be construed as a potential conflict of interest.

## Publisher’s Note

All claims expressed in this article are solely those of the authors and do not necessarily represent those of their affiliated organizations, or those of the publisher, the editors and the reviewers. Any product that may be evaluated in this article, or claim that may be made by its manufacturer, is not guaranteed or endorsed by the publisher.

## Supplementary Material

Supplementary File 1 The methods of protein isolation.

Supplementary Figure 1 Factors associated with recurrence-free survival (RFS) in univariable **(A)** and multivariable **(B)** Cox regression analysis in all the studied patients. **(A)** pCR status, ER positivity, PR positivity and HER2 positivity were associated with improved RFS, while higher pre-NAC clinical T and clinical N stages, mastectomy and ALND were associated with worse RFS in the univariable Cox regression analysis. **(B)** pCR status, pre-NAC clinical T and clinical N stages, ER positivity and axillary surgery were independent prognostic factors for RFS. ALND, axillary lymph node dissection; BCS, breast-conserving surgery; CI, confidence interval; ER, es-trogen receptor; HR, hormone receptor; HR*, hazard ratio; HER2, human epidermal growth factor receptor 2; PR, progesterone receptor; SLNB, sentinel lymph node biopsy; TNBC, triple-negative breast cancer.

Supplementary Figure 2 Subgroup analysis by patient and tumor characteristics of the prognostic value of pCR status for recurrence-free survival. The prognosis of pCR patients was better than that of the non-pCR patients across all subgroups. ALND, axillary lymph node dissec-tion; BCS, breast-conserving surgery; CI, confidence interval; ER, estrogen receptor; HR, hormone receptor; HR*, hazard ratio; HER2, human epidermal growth factor receptor 2; PR, progesterone receptor; SLNB, sentinel lymph node biopsy; TNBC, triple-negative breast cancer.

Supplementary Figure 3 The biological functions of the up-regulated differentially expressed proteins (DEPs). **(A)** Gene Ontology (GO) enrichment analysis of the DEPs. **(B)** Kyoto Encyclopedia of Genes and Genomes (KEGG) enrichment analysis of the DEPs. BP, biological process; CC, cellular component; Event_f, event free; MF, molecular function.

Supplementary Figure 4 The biological functions of the down-regulated differentially expressed proteins (DEPs). **(A)** Gene Ontology (GO) enrich-ment analysis of the DEPs. **(B)** Kyoto Encyclopedia of Genes and Genomes (KEGG) enrichment analysis of the DEPs. BP, biological process; CC, cellular component; Event_f, event free; MF, molecular function.
