# Supplementary material for: Markers Associated With Tumor Recurrence in Patients With Breast Cancer Achieving a Pathologic Complete Response After Neoadjuvant Chemotherapy
Source: Front Oncol. 2022 Apr 20;12:860475. doi: 10.3389/fonc.2022.860475 (PMC9067275; doi:10.3389/fonc.2022.860475)
Supplement: Supplementary File 1 — The methods of protein isolation. [file DataSheet_1.zip › Supplementary Files/Supplementary File S1.docx]

Label free – Methods

1. Total Protein Extraction [1]

The sample was dewaxed with octane, and then hydrated with graded ethanol. After hydration, the sample was washed twice with phosphate buffered saline (PBS). After removing the PBS solution, an appropriate amount of protein lysate (4% sodium dodecyl sulfate [SDS], 100 mM tris(hydroxymethyl) aminomethane [Tris]) was added and incubated at 95°C for 10 minutes at room temperature, mixed by shaking, and sonicated in an ice-water bath for 5 minutes. The samples were de-crosslinked with a refractive index at 95°C for 60 min, then reduced by adding appropriate amount of tris(2-carboxyethyl) phosphine (TCEP), and alkylated in chloroacetamide (CCA) at 95°C for 5min. The samples were sequentially centrifuged at 12000 g at 4°C for 15 min. Collecting the supernatant and adding 4 times volume of pre-cooling acetone at -20°C, and precipitated it at -20°C for at least 4h. Centrifuging at 12000 g for 15 min at 4°C . Collecting the precipitate and air drying. Appropriate amount of protein solution (8 M urea, 100 mM triethylammonium bicarbonate [TEAB], pH = 8.5) was added to dissolve the protein pellet.

1. Trypsin treatment [2]

Each protein sample was taken and the volume was made up to 100 μL with DB lysis buffer (8 M Urea, 100 mM TEAB, pH 8.5), trypsin and 100 mM TEAB buffer were added, sample was mixed and digested at 37 °C for 4 h. Then trypsin and CaCl_2_ were added digested overnight. Formic acid was mixed with digested sample, adjusted pH under 3, and centrifuged at 12000 g for 5 min at room temperature. The supernatant was slowly loaded to the C18 desalting column, washed with washing buffer (0.1% formic acid, 3% acetonitrile) 3 times, then added elution buffer (0.1% formic acid, 70% acetonitrile). The eluents of each sample were collected and lyophilized.

1. LC-MS/MS Analysis

Mobile phase A (100% water, 0.1% formic acid) and B solution (80% acetonitrile, 0.1% formic acid) were prepared. The lyophilized powder was dissolved in 10 μL of solution A, centrifuged at 14000 g for 20 min at 4°C, and 1 μg of the supernatant was injected into a home-made C18 Nano-Trap column (4.5cm×75 μm, 3 μm). Peptides were separated in a home-made analytical column (15 cm×150 μm, 1.9 μm), using a linear gradient elution as listed in Table 1. The separated peptides were analyzed by Q ExactiveTM HF-X mass spectrometer (Thermo Fisher), with ion source of Nanospray Flex™（ESI）, spray voltage of 2.1 kV and ion transport capillary temperature of 320°C. Full scan range from m/z 350 to 1500 with resolution of 60000 (at m/z 200), an automatic gain control (AGC) target value was 3×${10}^{6}$ and a maximum ion injection time was 20 ms. The top 40 precursors of the highest abundant in the full scan were selected and fragmented by higher energy collisional dissociation (HCD) and analyzed in MS/MS, where resolution was 15000 (at m/z 200), the automatic gain control (AGC) target value was 5×${10}^{4}$, the maximum ion injection time was 45 ms, a normalized collision energy was set as 27%, an intensity threshold was 2.2×${10}^{4}$, and the dynamic exclusion parameter was 20 s. The raw data of MS detection was named as “.raw”.

Table 1 Liquid chromatography elution gradient table

| Time | flow rate | mobile phase A | mobile phase B |
| --- | --- | --- | --- |
| (min) | (nL/min) | (%) | (%) |
| 0 | 600 | 94 | 6 |
| 2 | 600 | 90 | 10 |
| 45 | 600 | 70 | 30 |
| 48 | 600 | 65 | 35 |
| 50 | 600 | 50 | 50 |
| 51 | 600 | 0 | 100 |
| 60 | 600 | 0 | 100 |
| 60.5 | 600 | 95 | 5 |
| 61.5 | 600 | 95 | 5 |
| 62 | 600 | 5 | 95 |
| 67 | 600 | 5 | 95 |
| 70 | 600 | 95 | 5 |
|  |  |  |  |

1. Data analysis
   1. The identification and quantitation of protein

The resulting spectra from each fraction were searched separately against UniProt (homo_sapiens_uniprot_2020_7_2.fasta [192320 sequences]) database by the search engines: Proteome Discoverer 2.2 (PD 2.2, Thermo). The search parameters are set as follows: mass tolerance for precursor ion was 10 ppm and mass tolerance for product ion was 0.02 Da. Carbamidomethyl was specified in PD 2.2 as fixed modifications. Oxidation of methionine (M) and acetylation of the N-terminus were specified in PD 2.2 as variable modifications. A maximum of 2 missed cleavage sites were allowed.

The identified protein contains at least 1 unique peptide with FDR no more than 1.0%. Proteins containing similar peptides that could not be distinguished by MS/MS analysis were identified as a same protein group. Precursor ion was quantified by label-free quantification method based on intensity. The protein quantitation results were statistically analyzed by Mann-Whitney Test, for proteins whose quantitation significantly different between experimental and control groups, (p < 0.05 and FC > 1.2 or FC < 0.83 [fold change, FC]), were defined as differentially expressed proteins (DEPs).

- 1. The functional analysis of the DEPs

Gene Ontology (GO) analysis was conducted using the interproscan-5 program against the non-redundant protein database (including Pfam, PRINTS, ProDom, SMART, ProSiteProfiles, PANTHER) [3], the databases of KEGG (Kyoto Encyclopedia of Genes and Genomes) were used to analyze the protein family and pathway. The enrichment pipeline [5] was used for enrichment analysis of GO and KEGG.

**Abbreviations**

AGC, Automatic gain control; CAA, Chloroacetamide; DEP, Differentially expressed proteins; FC, Fold change; FDR, False discovery rates; GO, Gene Ontology; HCD, Higher energy collisional dissociation; KEGG, Kyoto Encyclopedia of Genes and Genomes; LC-MS, Liquid chromatography-mass spectrometry; PBS, Phosphate buffered saline; SDS, Sodium dodecyl sulfate; TCEP, Tris(2-carboxyethyl) phosphine; TEAB, Triethylammonium bicarbonate; TRIS, Tris(hydroxymethyl)aminomethane.

**Reference:**

[1] Buczak K, Kirkpatrick J M, Truckenmueller F, et al. Spatially resolved analysis of FFPE tissue proteomes by quantitative mass spectrometry[J]. Nature Protocols, 2020:1-24.

[2] Zhang H, Liu T, Zhang Z, et al. Integrated Proteogenomic Characterization of Human High-Grade Serous Ovarian Cancer[J]. Cell, 2016.

[3] Jones P, Binns D, Chang H Y, et al. InterProScan 5: genome-scale protein function classification[J]. Bioinformatics, 2014, 30(9): 1236-1240.

[4] Huang D W, Sherman B T, Lempicki R A. Bioinformatics enrichment tools: paths toward the comprehensive functional analysis of large gene lists[J]. Nucleic Acids Research, 2009, 37(1): 1-13.

[5] Franceschini A, Szklarczyk D, Frankild S, et al. STRING V9.1: Protein-Protein Interaction Networks, with Increased Coverage and Integration[J]. Nucleic Acids Research, 2012, 41(D1).
