# Supplementary material for: Markers Associated With Tumor Recurrence in Patients With Breast Cancer Achieving a Pathologic Complete Response After Neoadjuvant Chemotherapy
Source: Front Oncol. 2022 Apr 20;12:860475. doi: 10.3389/fonc.2022.860475 (PMC9067275; doi:10.3389/fonc.2022.860475)
Supplement: Supplementary File 1 — The methods of protein isolation. [file DataSheet_1.zip › Supplementary Files/Supplementary Table 1.docx]

Table S1. Clinicopathological characteristics of the pCR and non-pCR patients

| Characteristics | pCR  (n=420) | non-pCR  (n=1,437) |
| --- | --- | --- |
| **Age at diagnosis** |  |  |
| (median, IQR) | 48 (41-55) years | 45 (41-55) years |
| **Age** |  |  |
| ≤ 50 | 239 (56.9) | 872 (60.7) |
| > 50 | 181 (43.1) | 565 (39.3) |
| **Clinical T stage** |  |  |
| T1 | 73 (17.4) | 86 (6.0) |
| T2 | 248 (59.0) | 658 (45.8) |
| T3 | 47 (11.2) | 336 (23.4) |
| T4 | 27 (6.4) | 207 (14.4) |
| Unknown | 25 (6.0) | 150 (10.4) |
| **Clinical N stage** |  |  |
| N0 | 142 (33.8) | 185 (12.9) |
| N1 | 134 (31.9) | 380 (26.4) |
| N2 | 82 (19.5) | 479 (33.3) |
| N3 | 37 (8.8) | 208 (14.5) |
| Unknown | 25 (6.0) | 185 (12.9) |
| **Clinical TNM stage** |  |  |
| I | 37 (8.8) | 23 (1.6) |
| II | 216 (51.4) | 388 (27.0) |
| III | 141 (33.6) | 838 (58.3) |
| Unknown | 26 (6.2) | 188 (13.1) |
| **Histological type** |  |  |
| Invasive cancer non-specified | 263 (62.6) | 1,207 (84.0) |
| Others | 18 (4.3) | 86 (6.0) |
| Unknown | 139 (33.1) | 144 (10.0) |
| **Histological grade** |  |  |
| I | 3 (0.7) | 25 (1.7) |
| II | 126 (30.0) | 560 (39.0) |
| III | 168 (40.0) | 381 (26.5) |
| Unknown | 123 (29.3) | 471 (32.8) |
| **ER** |  |  |
| Negative | 225 (53.6) | 435 (30.3) |
| Positive | 190 (45.2) | 922 (64.2) |
| Unknown | 5 (1.2) | 80 (5.6) |
| **PR** |  |  |
| Negative | 239 (56.9) | 532 (37.0) |
| Positive | 163 (38.8) | 798 (55.5) |
| Unknown | 18 (4.3) | 107 (7.4) |
| **HER2** |  |  |
| Negative | 155 (36.9) | 800 (55.7) |
| Positive | 255 (60.7) | 508 (35.4) |
| Unknown | 10 (2.4) | 129 (9.0) |
| **Ki67** |  |  |
| < 20% | 48 (11.4) | 269 (18.7) |
| ≥ 20% | 356 (84.8) | 1,055 (73.4) |
| Unknown | 16 (3.8) | 113 (7.9) |
| **Molecular subtype** |  |  |
| HR+/HER2- | 77 (18.3) | 609 (42.4) |
| HER2+ | 255 (60.7) | 508 (35.4) |
| TNBC | 77 (18.3) | 184 (12.8) |
| Unknown | 11 (2.6) | 136 (9.5) |
| **NAC regimen** |  |  |
| E / T / E+T | 185 (44.0) | 973 (67.7) |
| Others | 234 (55.7) | 436(30.3) |
| Unknown | 1 (0.2) | 28 (1.9) |
| **Breast surgery** |  |  |
| BCS | 125 (29.8) | 175 (12.2) |
| Mastectomy | 291 (69.3) | 1,239 (86.2) |
| Unknown | 4 (1.0) | 23 (1.6) |
| **Axillary surgery** |  |  |
| SLNB | 176 (41.9) | 157 (10.9) |
| ALND+/-SLNB | 238 (56.7) | 1,272 (88.5) |
| Unknown | 6 (1.4) | 8 (0.6) |
| **Tumor recurrence** |  |  |
| No | 383 (91.2) | 929 (64.6) |
| Yes | 32 (7.6) | 444 (30.9) |
| Unknown | 5 (1.2) | 64 (4.5) |
| **Recurrent or metastatic lesions** |  |  |
| Local-regional relapse | 17 (4.0) | 180 (12.4) |
| Liver | 3 (0.7) | 56 (3.9) |
| Lung | 4 (1.0) | 66 (4.6) |
| Bone | 3 (0.7) | 89 (6.2) |
| Brain | 5 (1.2) | 21 (1.5) |
| Soft tissue | 1 (0.2) | 24 (1.7) |
| Ovary | 0 (0) | 1 (0.1) |
| Multi-organs | 8 (1.9) | 90 (6.3) |
| Unknown |  | 1 (0.4) |

AC, adjuvant chemotherapy; ALND, axillary lymph node dissection; BCS, breast-conserving surgery; E, antharcycline; ER, estrogen receptor; HER2, human epidermal growth factor receptor 2; IQR, interquartile range; NAC, neoadjuvant chemotherapy; PR, progesterone receptor; SLNB, sentinel lymph node biopsy; T, taxine
