# Supplementary material for: Markers Associated With Tumor Recurrence in Patients With Breast Cancer Achieving a Pathologic Complete Response After Neoadjuvant Chemotherapy
Source: Front Oncol. 2022 Apr 20;12:860475. doi: 10.3389/fonc.2022.860475 (PMC9067275; doi:10.3389/fonc.2022.860475)
Supplement: Supplementary File 1 — The methods of protein isolation. [file DataSheet_1.zip › Supplementary Files/Supplementary Table 2.docx]

| **Table S2.** 43 up-regulated and 84 down-regulated differentially expressed proteins and their corresponding genes. | | |
| --- | --- | --- |
|  | **DEPs** | **DEGs** |
| **up-regulated** | Q3KQU3 | MAP7D1 |
|  | A0A0G2JMX7 | MAPT |
|  | Q8IXW6 | DPYSL3 |
|  | Q13784 | APOA4 |
|  | A0A087X256 | WASHC4 |
|  | Q96T51 | RUFY1 |
|  | Q8IVL6 | P3H3 |
|  | Q96HY7 | DHTKD1 |
|  | O60884 | DNAJA2 |
|  | A0A494BZV2 | MPRIP |
|  | Q9GZP4 | PITHD1 |
|  | F8W6G1 | NRBP1 |
|  | Q8ND56 | LSM14A |
|  | P84098 | RPL19 |
|  | Q86W92 | PPFIBP1 |
|  | Q32P28 | P3H1 |
|  | B7Z6F7 |  |
|  | Q9NU22 | MDN1 |
|  | P02042 | HBD |
|  | Q9Y376 | CAB39 |
|  | H0YKD8 | RPL28 |
|  | P00813 | ADA |
|  | A8K586 |  |
|  | O14910 | LIN7A |
|  | Q5T5P2 | KIAA1217 |
|  | A8K7Z3 |  |
|  | Q96AY3 | FKBP10 |
|  | B2RDQ3 |  |
|  | A0A384NKM6 | APOH |
|  | Q15363 | TMED2 |
|  | B4DUV1 |  |
|  | O75190 | DNAJB6 |
|  | A0A650F0N3 |  |
|  | Q9H3N1 | TMX1 |
|  | B2R673 |  |
|  | A0A024RBH2 | CKAP4 |
|  | Q6DEN2 | DPYSL3 |
|  | A0A087WUT6 | EIF5B |
|  | A8K4K1 |  |
|  | X6R8A1 | CTSA |
|  | A0A1U9X972 |  |
|  | A0A024R850 | MRRF |
|  | P19525 | EIF2AK2 |
| **down-regulated** | Q13427 | PPIG |
|  | A4D2P0 | RAC1 |
|  | P07384 | CAPN1 |
|  | B2ZZ89 | SPTBN1 |
|  | A8K3S1 |  |
|  | P47224 | RABIF |
|  | P37837 | TALDO1 |
|  | A0A023I7V4 | ATP6 |
|  | A8K940 |  |
|  | A0A384NL22 | PSMB3 |
|  | U3KQL2 | ACYP2 |
|  | P12081 | HARS1 |
|  | B2RE76 | CHMP2B |
|  | G8JLB6 | HNRNPH1 |
|  | O14920 | IKBKB |
|  | A0A3B3IUD7 | PDCD11 |
|  | B8ZWD9 | DBI |
|  | B5BUI8 | DUSP3 |
|  | B2RB23 |  |
|  | Q9BTV4 | TMEM43 |
|  | P04040 | CAT |
|  | H9STE0 | COX2 |
|  | A0A0A0MTH3 | ILK |
|  | A0A024R2K1 | RAB5A |
|  | Q96EK4 | THAP11 |
|  | A0A0M4FNU3 | ALDOA |
|  | P62330 | ARF6 |
|  | A0A024RCM3 | hCG_2005638 |
|  | Q9C0I1 | MTMR12 |
|  | Q9Y3E8 |  |
|  | D3DU01 | TMEM49 |
|  | O43818 | RRP9 |
|  | P13498 | CYBA |
|  | O15173 | PGRMC2 |
|  | O95140 | MFN2 |
|  | J3KPD6 | HCK |
|  | A0A024R539 | LOC51035 |
|  | Q99567 | NUP88 |
|  | P42566 | EPS15 |
|  | A0A024R5X2 | hCG_2001986 |
|  | A0A0D9SFG6 | CYTH2 |
|  | Q86VS8 | HOOK3 |
|  | Q8IYB8 | SUPV3L1 |
|  | A8K3W7 |  |
|  | P52948 | NUP98 |
|  | P30622 | CLIP1 |
|  | Q5HYL4 | DKFZp686E1893 |
|  | B8ZZ87 | MZT2B |
|  | A0A0S2Z5M8 | ELAC2 |
|  | D3DUE7 | N-PAC |
|  | Q6ZWT7 | MBOAT2 |
|  | A0A024R2M6 | ACAA1 |
|  | Q9BVL2 | NUP58 |
|  | L7N2F9 |  |
|  | Q5W009 | RBM17 |
|  | P50238 | CRIP1 |
|  | J3QL71 | SCRN2 |
|  | Q96P48 | ARAP1 |
|  | A0A384P5U2 |  |
|  | A0A5F9ZHH3 | SEPTIN4 |
|  | Q9BT23 | LIMD2 |
|  | O15357 | INPPL1 |
|  | Q02252 | ALDH6A1 |
|  | Q9UQ13 | SHOC2 |
|  | O43290 | SART1 |
|  | A0A499FIZ0 | WDR26 |
|  | A0A024R8L7 | ACOX1 |
|  | P39059 | COL15A1 |
|  | Q13405 | MRPL49 |
|  | P57772 | EEFSEC |
|  | Q9NW15 | ANO10 |
|  | E7EPT4 | NDUFV2 |
|  | P36507 | MAP2K2 |
|  | A6NI72 | NCF1B |
|  | P22059 | OSBP |
|  | A0A5C2GFJ1 |  |
|  | O75663 | TIPRL |
|  | F8VXC8 | SMARCC2 |
|  | A0A5F9ZHN9 | ALDH3A2 |
|  | A0A024QZB4 | hCG_1993905 |
|  | Q9BRX8 | PRXL2A |
|  | P07327 | ADH1A |
|  | B1AKJ6 | OSBPL9 |
|  | A0A024R2B6 | SERPINB5 |
| DEPs, differentially expressed proteins; DEGs, differentially expressed genes | | |
