# Supplementary figures and images for: Markers Associated With Tumor Recurrence in Patients With Breast Cancer Achieving a Pathologic Complete Response After Neoadjuvant Chemotherapy
Source: Front Oncol. 2022 Apr 20;12:860475. doi: 10.3389/fonc.2022.860475 (PMC9067275; doi:10.3389/fonc.2022.860475)

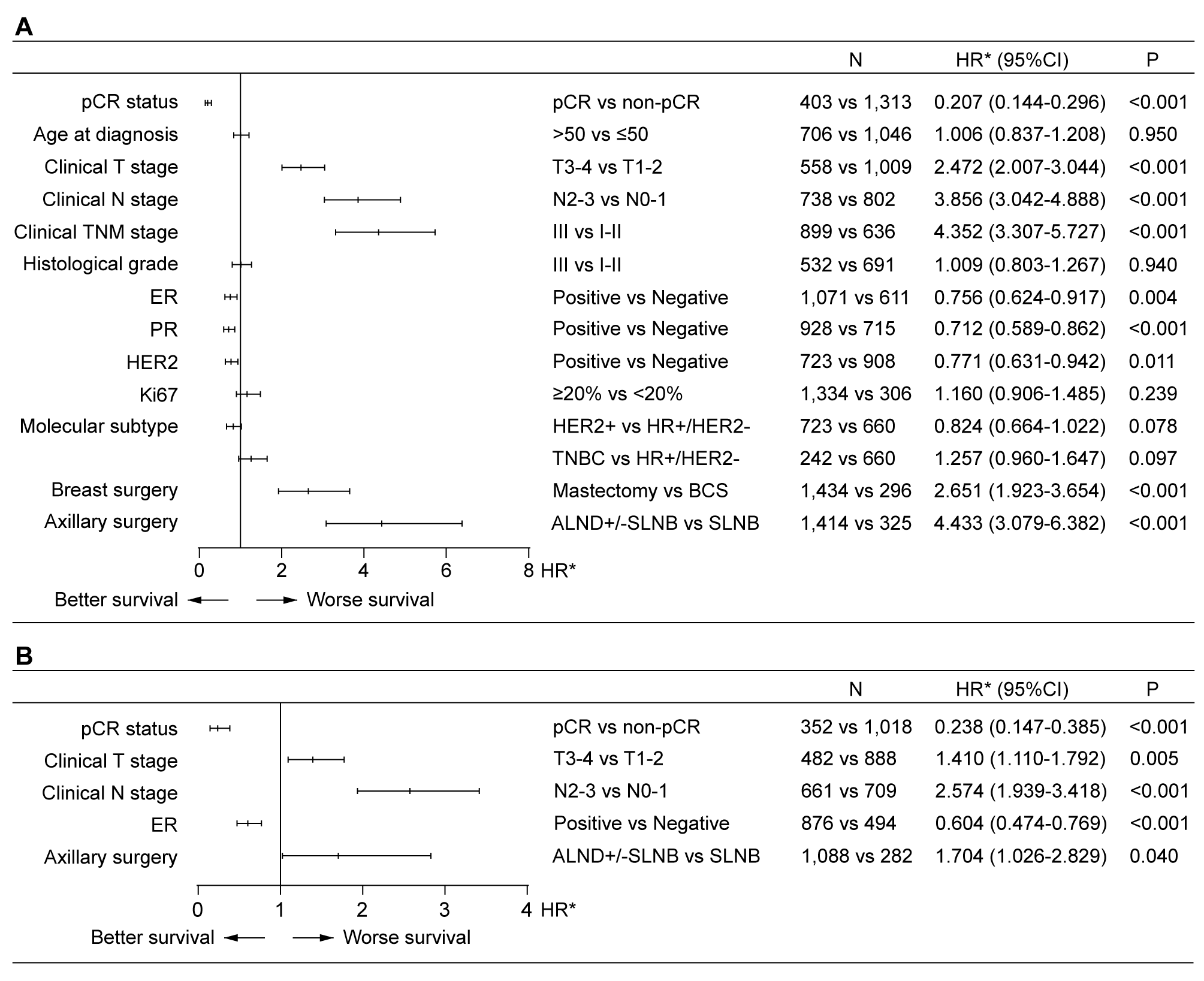

Supplement: Supplementary File 1 — The methods of protein isolation. [file DataSheet_1.zip › Supplementary Files/Supplementary Figure 1.tif]

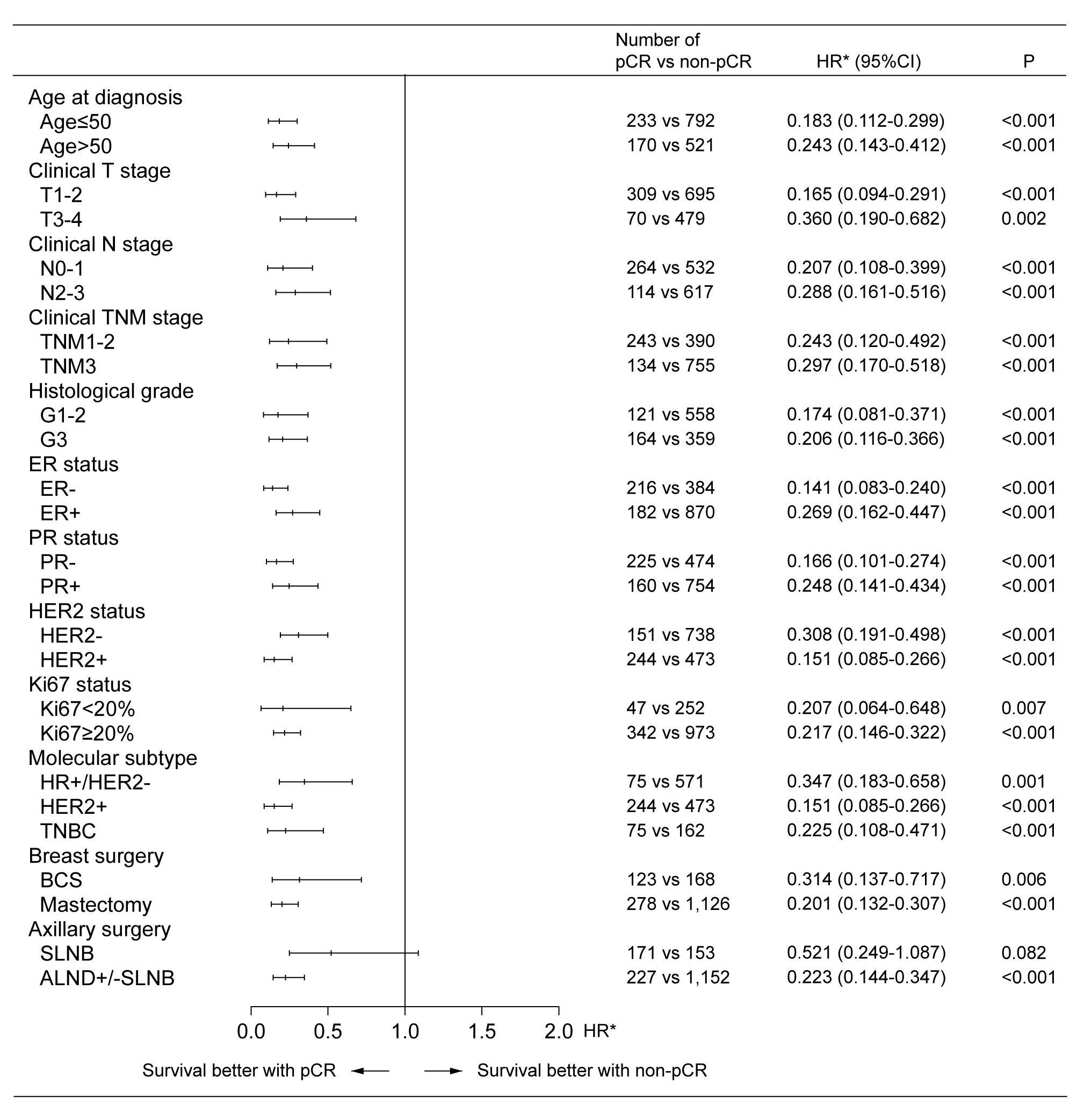

Supplement: Supplementary File 1 — The methods of protein isolation. [file DataSheet_1.zip › Supplementary Files/Supplementary Figure 2.tif]

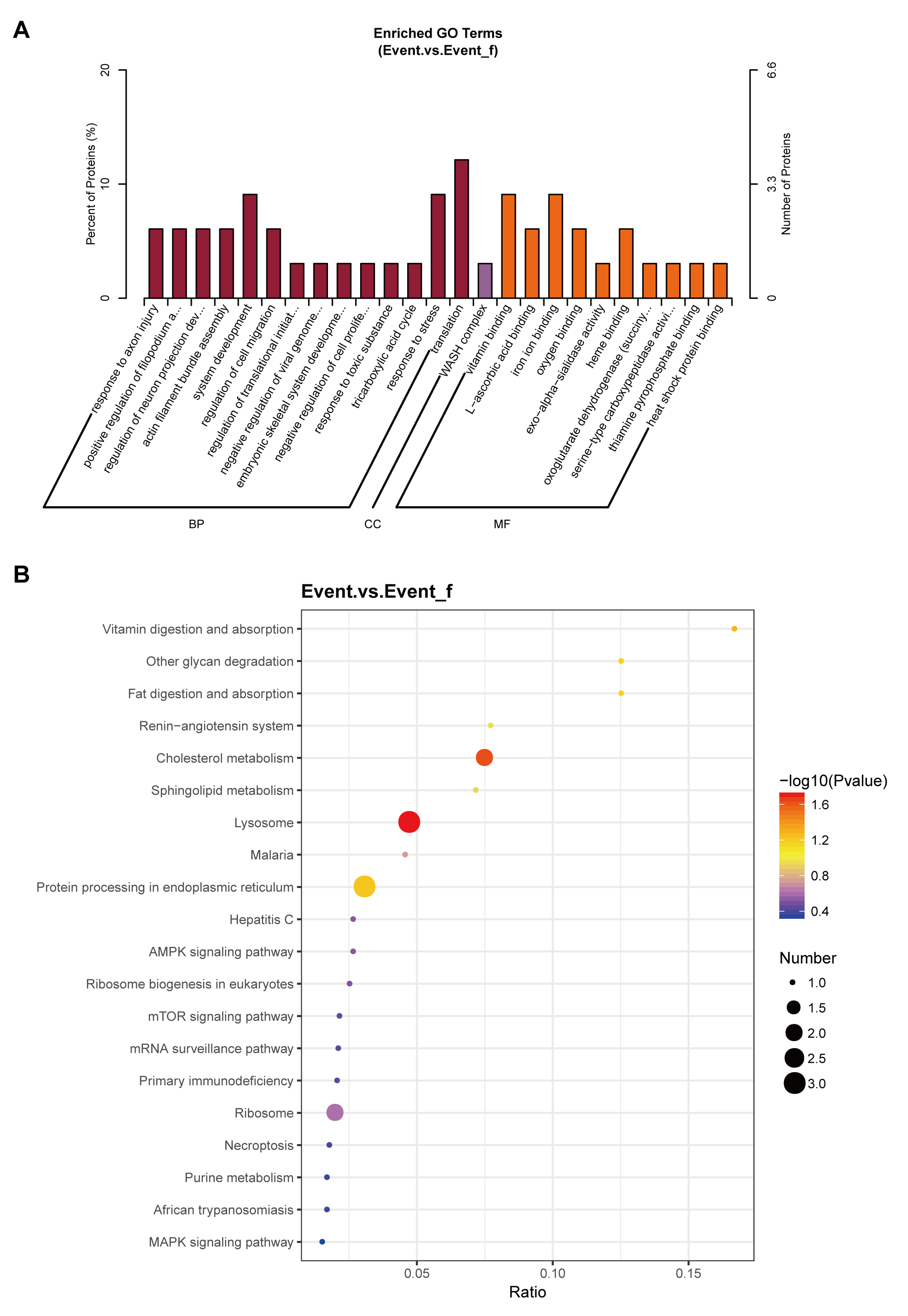

Supplement: Supplementary File 1 — The methods of protein isolation. [file DataSheet_1.zip › Supplementary Files/Supplementary Figure 3.tif]

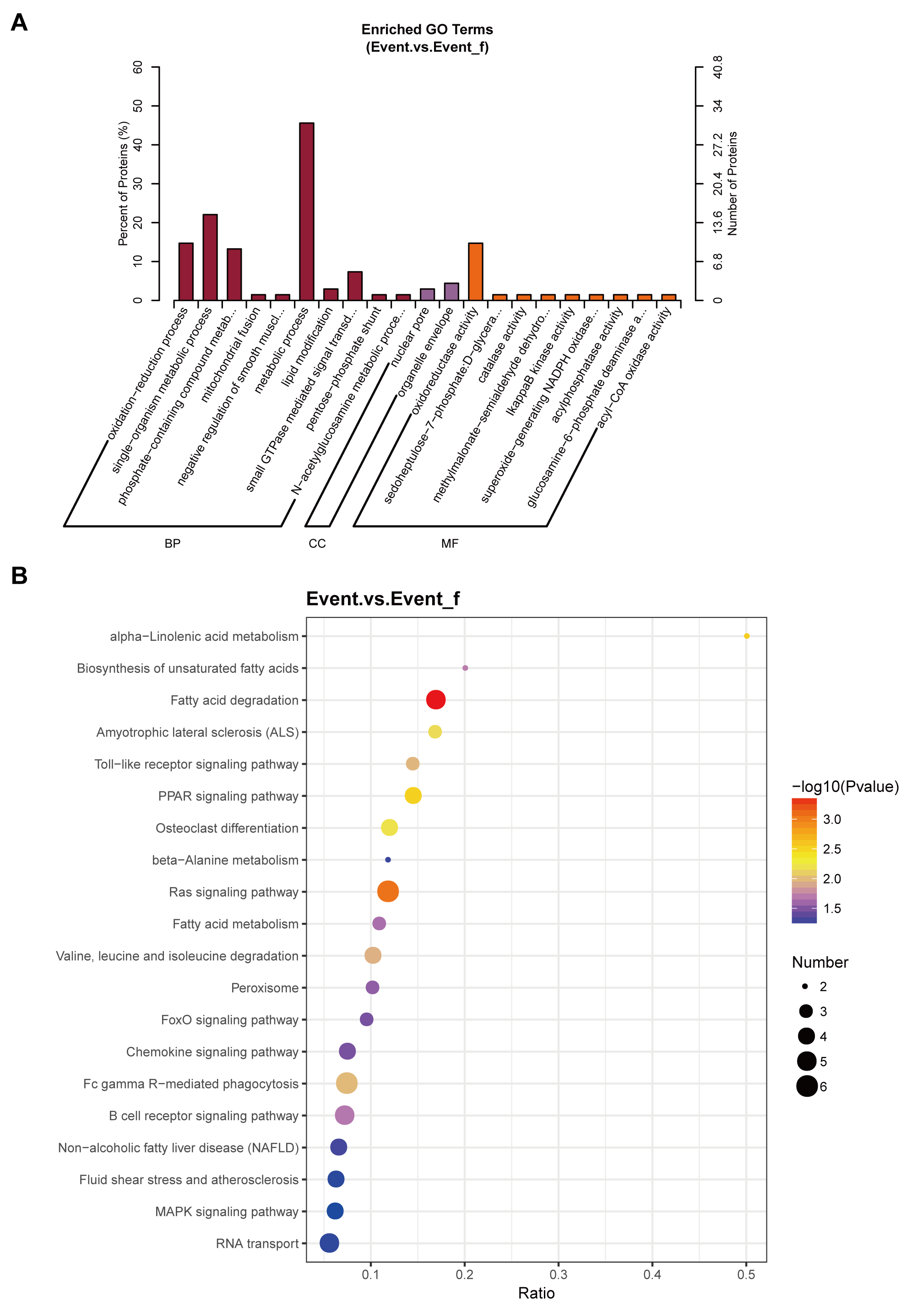

Supplement: Supplementary File 1 — The methods of protein isolation. [file DataSheet_1.zip › Supplementary Files/Supplementary Figure 4.tif]
